# Influence of Halide Solutions on Collagen Networks: Measurements of Physical Properties by Atomic Force Microscopy 

**DOI:** 10.1155/2016/4956756

**Published:** 2016-09-18

**Authors:** Birgit Spitzer-Sonnleitner, André Kempe, Maximilian Lackner

**Affiliations:** ^1^Paracelsus Society of Balneology and Iodine Research, Kurpromenade 1, 4540 Bad Hall, Austria; ^2^University of Applied Sciences Upper Austria, Campus Linz, FH OÖ Studienbetriebs GmbH, Franz-Fritsch-Straße 11/Top 3, 4600 Wels, Austria; ^3^University of Applied Sciences Technikum Wien, Hoechstaedtplatz 6, 1200 Vienna, Austria

## Abstract

The influence of aqueous halide solutions on collagen coatings was tested. The effects on resistance against indentation/penetration on adhesion forces were measured by atomic force microscopy (AFM) and the change of Young's modulus of the coating was derived. Comparative measurements over time were conducted with halide solutions of various concentrations. Physical properties of the mesh-like coating generally showed large variability. Starting with a compact set of physical properties, data disperse after minutes. A trend of increase in elasticity and permeability was found for all halide solutions. These changes were largest in NaI, displaying a logical trend with ion size. However a correlation with concentration was not measured. Adhesion properties were found to be independent of mechanical properties. The paper also presents practical experience for AFM measurements of soft tissue under liquids, particularly related to data evaluation. The weakening in physical strength found after exposure to halide solutions may be interpreted as widening of the network structure or change in the chemical properties in part of the collagen fibres (swelling). In order to design customized surface coatings at optimized conditions also for medical applications, halide solutions might be used as agents with little impact on the safety of patients.

## 1. Introduction

Collagen I was used for the experiments, as it is widely distributed in the body, found in the extracellular structures of the connective tissue, skin, blood vessels, tendons, cartilages, and several eye tissues. It is necessary to stabilize the tissues of the body and it is also used for force transmission [1, 2].

The basic unit of collagen is the tropocollagen molecule, consisting of 3 equally long polypeptide chains. These tropocollagen molecules are self-assembled into 10 to 300 nm sized fibrils with regularly staggered ends, and these fibrils are agglomerated into 0.5 to 3 *μ*m thick collagen fibres. There are also nonhelical gaps between the tropocollagens which play some role in hydration and swelling.

The arrangement of the fibrils determines the biological function of the tissues. The parallel arrangement in the tendons is responsible for strength transfer, the collagen network of the skin contributes to its tensile strength, and the properties of the vitreous body of the eye are caused by a light-permeable fibril net.

The special properties of collagen favour its application as a biomaterial for tissue engineering and in regenerative medicine. Collagen materials are biocompatible, biodegradable, easily available, and highly versatile; they have been tested and envisaged as biomaterial for tissue engineering, for example, for drug delivery [3] or scaffolds [4], implantable medical devices, biocompatible coatings, and biomaterials for biotechnology.

All of these as well as the use as adhesive cover for biomedical experiments implemented on the petri dishes studied here imply the importance of understanding the physical properties of this material.

In this work, the effect of halides on the mechanical properties of collagen (in the form of a spin coated network) was investigated. The general properties of collagen have been investigated and published by several authors; see, for example, [5]. As shown in Table 1, absolute values vary roughly by the factor 10, thus proofing a consistent knowledge base for single molecules and fibres. Generally again in elasticity when stepping from molecule to the fibre compound is observed (Table 1).

Atomic force microscopy (AFM) was used to probe the tissues due to its high spatial and temporal resolution.

This paper adds to the body of collagen knowledge by addressing changes in the mechanical properties by treatment with aqueous solutions of halides.

## 2. Materials and Methods

### 2.1. Materials

#### 2.1.1. Collagen

The elastic properties of collagen type I fibres were examined in bulk, assembled to networked structures by an industrial spin coating process and crosslinked to a certain degree. 11 commercial petri dishes (CELLCOAT cell culture dishes made from polystyrene, Greiner Bio-One, Germany) were studied.

#### 2.1.2. Solutions

Based on the work performed in [6–10] NaCl, NaI, and NaBr were chosen to investigate the effect of the halide anion on a collagen network.

#### 2.1.3. Isotonic NaCl Solution

The authors used isotonic NaCl solution (9 g/L) for medical infusion (Fresenius, Germany) as a reference point with supposedly minimal influence on the substrate.

#### 2.1.4. Halide Solutions

Comparative measurements were conducted with halide solutions with varying salt concentrations in aqua bidest. (product number P07113-6 Ph.Eur., conductivity ≤5 *μ*S/cm; Fresenius, Germany), as shown in Table 2.

### 2.2. Atomic Force Microscopy (AFM)

CELLCOAT collagen type I cell culture dishes and test solutions were held at room temperature for several hours for thermal adaptation prior to the experiments. Dishes were fully covered with 20 mL of fluid, roughly creating a 3 mm thick film just at the start of time recording. After one minute the atomic force microscope sensor was immersed in the fluid and allowed 10 to 30 minutes of adaptation to thermal conditions. Measurements were started as soon as drift effects lessened to an negligible amount and performed until 190-minute time in immersion. Atomic force microscopy was performed with an AFM type 5400 (Agilent Technologies, California), equipped with a 3-piezo scanner with a lateral range of 98 *μ*m and a vertical range of 7.8 *μ*m, capable of performing force mapping and real time calculation of indentation depth, adhesion force, and Young's modulus. For each specimen one initial topography image was taken during adaptation time in order to have a measure for surface roughness. Afterwards the scanner was shifted to a new position and switched to force spectroscopy mode, then performing force volume point spectroscopy measurements at a speed of 1 *μ*m per second for vertical piezo movement during loading/unloading and a 9-second break between the 16 × 16 measurements arrayed on a square field of 2 × 2 *μ*m. The scanner was again shifted to a new position after completion of each force mapping volume, thus yielding a continuous row of 6 nanoindentation measurements on untouched collagen surface every minute.

Physical properties of the collagen coating were measured with contact mode AFM probes, (model MLCT-D, Bruker, California) using the nominal tip radius of 10 nm and nominal spring constant of 0.03 N/m for calculation. MLCT-D [11] was selected for best results after comparison with probes of lower and higher spring constant.

Each point spectrum measured consists of a force distance curve alike to the typical example shown in Figure 3, with the loading cycle depicted in blue and unloading shown in red. The indentation depth was read out from the loading curve; elasticity was calculated from the unloading curve, using the DMT model for elastic modulus [12]. Adhesion force was calculated from the first large peak in the unloading curve.

The correlation of indentation depth and modulus is qualitative, as well as the correlation of adhesion and indentation depth.

Measurement of all parameters had the lowest degree of scattering and the lowest modification trend performed in isotonic NaCl; this was used as a benchmark for the other experiments.

Each dataset measured was corrected by a constant factor to bring the initial mean of absolute values to the mean of the initial value measured for collagen under isotonic NaCl. For simple setups like Agilent 5400, to the authors' experience, this correction method is more precise than any effort to achieve absolute calibration; however it does not yield quantitative data.

### 2.3. Calculation of Modulus

Young's modulus, also known as the tensile modulus or elastic modulus, is the relation between stress and strain of an elastic, isotropic material [13]. It is therefore a measure for the material's response in elastic strain to a stress, imposed on a given area. Modulus was calculated with the DMT model [12], implemented as a component to PicoView 1.14 AFM analysis software.

Wenger et al. [14] state that variation in the Poisson ratio of single collagen fibrils due to anisotropy is negligible for the calculation of Young's modulus. However since in this study a mesh of fibrils was examined, a variety of contact geometries, angles, and hierarchical network connections of various fibrils must be expected which will also impose their error on *ν*. The authors expect that variation in Poisson's ratio as well as variation in contact surface occurred; however, it cannot be accounted for numerically.

## 3. Results and Discussion

### 3.1. Material Structure

**Figure 1:**
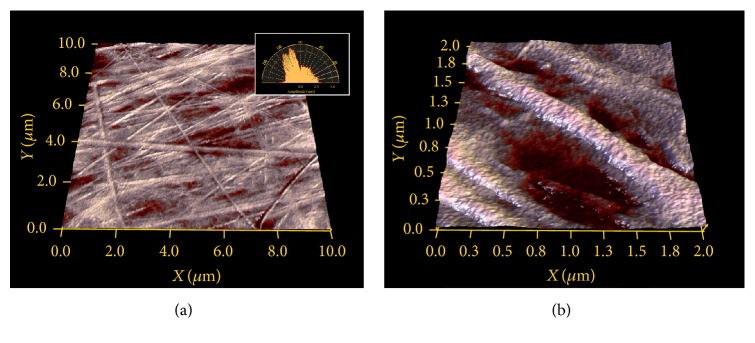
Topographical images of collagen tissue. (a) 10 *μ*m × 10 *μ*m image size, with angular spectrum showing a predominant direction around 120°; (b) 2 *μ*m × 2 *μ*m image size, with substructure on collagen fibre and network valley depicted in red.

Topographic imaging showed surfaces composed of a mesh of fibres of variable thickness and orientation; see Figure 1. Straightforward topographical feature analysis was first applied in order to determine if the morphologically heterogeneous-looking structure will or will not interfere with modulus values measured across the different spots on the mesh. Typically variations in material composition can only be deducted if topography does not play a role for the calculation model.

The texture direction index [15] with values of 0.45 ± 0.13 averaged for 11 specimens shows that many fibres are oriented in a dominant direction, where a value of 0 would mean fully parallel fibres and a value of 1 would indicate homogeneous distribution of directions [16]. The predominance of a specific direction is visualized in the angular spectrum in Figure 1(a). Roughness Analysis on image sizes of 2 *μ*m × 2 *μ*m (e.g., Figure 1(b)) reveals average root mean square (RMS) roughness of 24 nm ± 5.4 nm, which is just in the dimension of the sensor apex.

**Figure 2:**
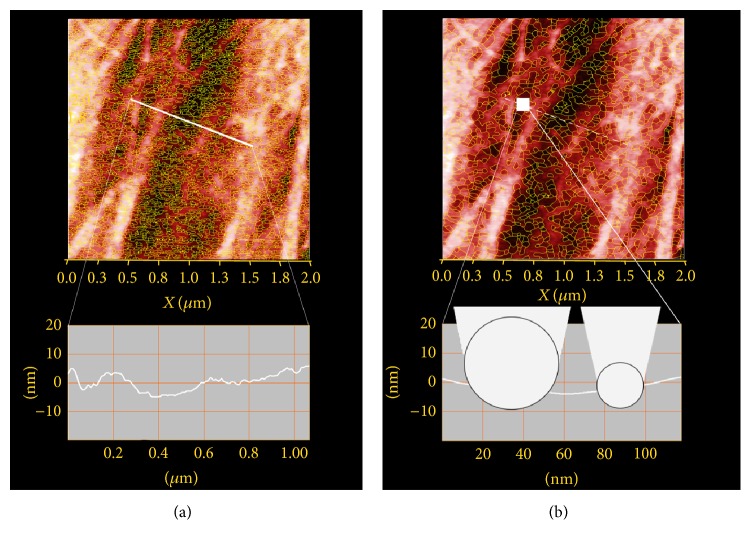
Topographical image of collagen tissue with local valley detection results marked in yellow; 2 *μ*m × 2 *μ*m image size; white line in (a) showing position of height profile; white box in (b) showing position of detailed profile. Local valley radius: (a) 10 nm/(b) 20 nm. Bottom left: height profile with exaggerated height values along the white line shown in top images. Bottom right: height profile section from the white box situated in one of the most extreme topographical pattern situations of the image and showing true proportions; herein depictions of sensor apexes with radiuses 10 nm and 20 nm in true dimensions and positioned at the average indentation depth measured.

In order to evaluate the potential for pore and trench structures to interfere with the geometry of the sensor apex, pore analysis by the watershed method was performed, using slope to detect local valleys on the surface. With a slope threshold set to 10%, the smoothing filter size was adjusted to yield all local valleys with a radius of 10 nm and 20 nm, respectively, taking account of apex sizes of the same dimensions (Figure 2).

Within the representative area of 4 *μ*m^2^, 5177 valleys with a 10 nm radius (Figure 2(a)) and 1235 valleys with a 20 nm radius (Figure 2(b)) were counted. The profile shows peak to peak roughness of 8.5 nm, including more shallow local minima. Local valley analysis revealed a maximum depth of 1.4 nm for valley structures with radius of 20 nm and 0.9 nm for the smaller ones. Local valleys were hence found to be in general much shallower than the dimension of the sensor apex. Therefore the effect of topography on the results of indentation measurement is negligible and the surface is assumed to be sufficiently flat to be examined as bulk material.

### 3.2. Force Measurements

**Figure 3:**
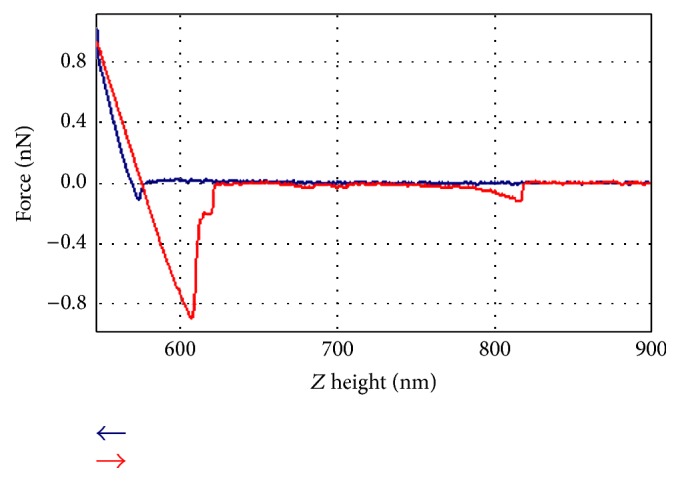
Typical force-distance graph. See text for details.

During each measurement cycle, indentation forces were raised constantly to 0.13 nN and then released at a constant rate. Representative force-distance curves showed typical interactions as responses of the collagen network to the indenter (Figure 3).

- Approach: as the cantilever approaches the surface, short-range forces such as Van Der Waals, electric, or capillary forces come to effect as soon as their range is surpassed by the probe. If an attractive force layer is present on the surface before the probe-sample contact, the tip might jump towards surface (snap-in). Since measurements were conducted in fluid, electrical or capillary effects are not present in the system, leaving possible attractive effects to Van Der Waals forces in short-range distance. For most force curves no or very little snap-in movement was observed, showing that the first attractive contact of tip and surface must have been occurring with very little deformation of the cantilever. If any adhesive interaction was taking place at this stadium of the force curve, it was hence not measured. However, envisioning the soft nature of the surface that consists of a cross mesh of long fibrils that are surrounded by aqueous fluid, it becomes conceivable that adhesive interaction may well have taken place while loosely bound fibrils floated towards the tip of the probe and adhered there without pulling it down. The authors interpret the passive nature of the approach as an indication for a soft surface without well-defined boundaries.
- Contact and indentation: sinking the probe into the surface took place in a phase of elastic deformation where both the probing cantilever and the surface were bent until the modulus of the collagen network surpassed the driving force of the indenter. This stop point was typically found within a deformation depth of 5 to 15 nm. At this depth the properties of the physically stable bulk material were measured.
- Retraction and adhesion: in most cases adhesive forces caused the material from the bulk collagen to attach to the sensor, pulling it downwards during retraction with variable forces sometimes as large as 0.8 nN. Strong adhesive forces typically reached out into the fluid of 50 to 100 nm beyond the touchdown point of the probe. This leads to the assumption that sticky material was pulled out of the surface together with the tip as it is typical for soft, easily deformable surfaces. This distance is read out from the first big peak of negative force, shown in red in Figure 3.
- Pull-back interactions often showed multistacked rip of dynamics and occurred in more than one snap-off event as depicted in the smaller negative force peaks in Figure 3. The release of the attached fibrils therefore happened in successive steps, reaching out as far as 300 nm over surface level. The authors assume that fibrils get picked up by the tip apex and get dragged up into the liquid, where attraction stress is raised to a maximum. At the snap-out point contact with the sensor is lost and the negative pull-back force becomes zero.

### 3.3. Nanomechanical Properties

A minimum number of 600 values per experiment have been collected during 90 minutes of measurements. 30-minute intervals in the beginning of each experiment are compared to 30-minute intervals at the end.

**Figure 4:**
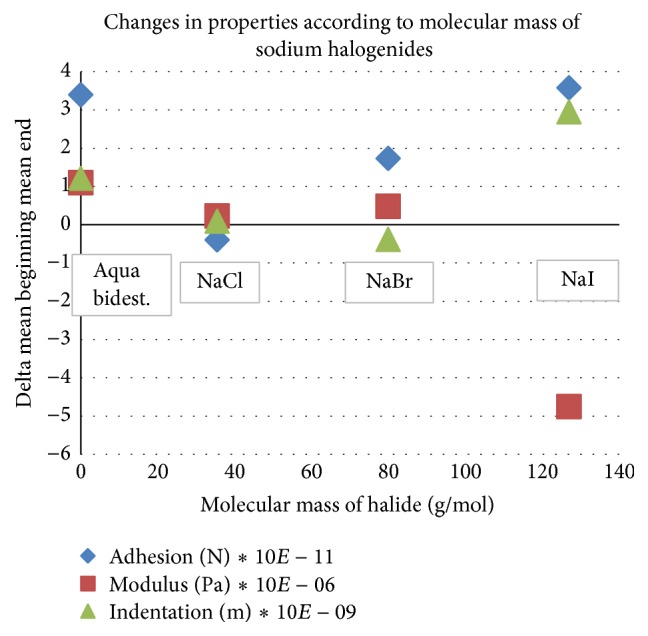
Overview on the change in nanomechanical properties, during exposure to halide solutions at a concentration of 0.15 mol/L.

Figure 4 shows the calculated changes in all 3 parameters, indentation depth, adhesion, and modulus between the mean values of each interval. Material change is the lowest for chloride, increased for bromide, and strongest for iodide solutions.

Aqua bidestillata displays comparably strong influence (Figure 4).

Indentation depth is the primary parameter for the measurement of material resistivity. The changes in indentation (green triangles) increase with increasing molecular weight of the halide ion. This means material weakening or softening after exposure to the larger ions occurs. A symmetrical decrease in the changes of Young's modulus depicted by the red quadrangles appears again in elasticity after halide exposure and is consistent with the indentation results.

Adhesion forces are a sum of chemical and physical interactions and in principal are not directly linked to indentation or modulus, because they occur after the depression phase of the indentation movement. Figure 4 reveals positive changes in adhesion for all solutions except NaCl and a positive trend together with the molecular weight of the ion.

**Figure 5:**
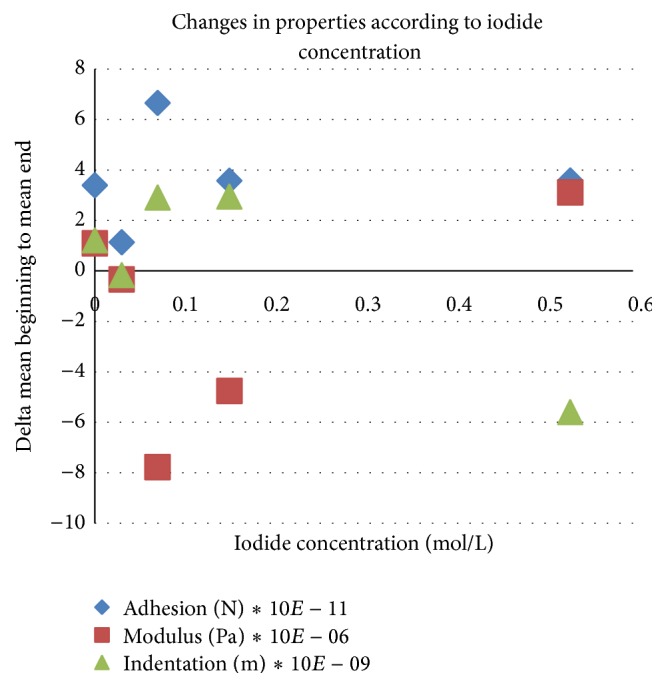
Overview on the change in nanomechanical properties, during exposure to iodide solutions at varied concentration.

Although the experimenter might expect a linear dependency between the concentration of ions and the material change described above, this was not confirmed by our experiment.  Figure 5 shows a more complicated result for variation in the concentration of iodide. Although exposure to iodide solutions at varied concentrations caused significant changes in the same direction as described for the isotonic concentration of 0.15 mol/L in most cases, no direct correlation of the material effect with concentration could be found. Trends would even turn around for the higher concentrations and for distilled water (Figure 5).

## 4. Discussion

Collagen is known to swell when in contact with water and it is known that the swelling can be enhanced and accelerated by the addition of various salts [6, 7]. The results of the AFM measurements clearly show the effect of the absence of salts and of the presence of salts at various concentrations.

Exposed to halide ion solutions, the collagen mesh showed dynamic behaviour and changes in its nanomechanical properties. The three halides studied, Cl^−^, Br^−^, and I^−^, have the same charge but different radii. Hence the charge density on the ions' surface varies, and one might expect the strongest interaction with Cl^−^ ions, which have the smallest radius and hence the largest charge density. This is not the case.

Comparing the changes in collagen mechanical properties under the influence of the different halides, a strong effect of the iodide ion and smaller influence of the lighter ions chloride and bromide become evident. The surface layer of the collagen shows different changes in modulus and indentation strength after exposure to the three halides. A systematic linear correlation of the change in resistance to compression (Young's modulus) with atomic weight of the halide or the corresponding ion radius was not found, although the overall picture points to the idea that ion size plays an important role. Neither changes in indentation depth nor moduli are linearly correlated.

However a linear increase in the change of adhesive strength between the mesh and the silicon nitride sensor tip was found, along with the increase in weight of the halide.

The effect of aqua bidest. is stronger than that of the light halides, for both compressive and adhesive properties only topped by iodide. The pure aquatic system is therefore regarded as a separate regime that cannot be taken as the baseline for halide testing, being nonphysiological in its nature. Iodide solutions clearly cause the highest change in adhesive strength and show largest change in the softening effects after exposure times of 90 minutes.

A more complex discussion has to be lead when looking at different concentrations. When exposed to iodide solutions of increasing concentration, changes in indentation depth, along with adhesion forces, show a trend up, which means that the distance in which tip and sample interact is enlarged. The effect is comparable to swelling which will soften the outer layer of the network, encouraging the collagen fibres to reach out farther into the liquid. The substance is therefore becoming less stable and stickier. Swelling, however, is inverted at some point around 0.07 mol/L. It is suggested that this nonlinear behaviour reflects a change in the modification of the molecular makeup of the collagen network and is inflicted by the chaotropic influence of the iodide ion as described by the Hofmeister series [17]. The Hofmeister series (lyotropic series) is a classification of ions in order of their ability to salt out or salt in proteins.

Modulus changes are first decreasing and then increasing with higher concentrations. Since an increase of modulus means less elasticity and more stiffness, the substance becomes softer as the surface layer of the substance adds volume and stickiness. Also for elasticity, the turning point of the Hofmeister development is found around 0.07 mol/L.

In summary the authors interpret the surface of the collagen mesh as a dynamic system that clearly responds to the influence of aqueous halide solutions.

The variability in pull-back force curves shows a range of interaction dynamics and force magnitudes, implying that the type and strength of interaction with a nanoscaled object largely depend on the spot of indentation. This variation can rather be explained by inhomogeneity of the multifibre system compared to simple topographic effects, because surface roughness is of much smaller dimensions than the apex radius of the probe.

Because exposure to iodide solutions with a systematic increase in molarity yielded a systematic but nonlinear pattern with a turning point around 0.07 mol/L for adhesion, indentation, and modulus dynamics, the authors interpret that the known swelling effect of collagen was observed in this study and its maximum lies at 0.07 mol/L, where a softer surface is correlated with a larger stretch range of molecular fibrils.

The authors envisage a fibrous network, consisting of an inner bulk of stable fibrils and an outer soft layer of loosely dangling fibrils, mostly adhering to each other due to Van Der Waals forces. The outer layer corresponds roughly to the indentation depth measured, between 5 and 15 nm thickness. It would contain collagen fibrils that are highly hydrated and not completely fixed to their neighbours. The higher the hydration force of the fluid becomes, the more easily these fibrils would be dragged away from the bulk material. The results presented here show that fibrils can be dragged out as far as 200 nm before adhesion to the retracting probe is broken.

Higher concentrations of ions change the outer layer, fibres get more dispersed in the aqueous solution, and their capacity of adhering to small objects such as a nanoprobe will largely increase, explaining the constantly increasing adhesion forces measured for all fluids. At the same time swelling of this outer layer will loosen its protective character as a soft cover on the inner network which is more stable and less elastic. While the probe sinks deeper into the outer layer, it reaches deeper into the stiffer bulk layer, which will result in measurement of an increased modulus.

To summarize, it could be shown that the sodium halides of Cl^−^, Br^−^, and I^−^, with the largest effects shown for iodide, are suitable for altering the properties of collagen.

## 5. Outlook

Swelling of collagen tissue could provide a promising route for engineering specific materials for medical use. The nontoxic nature of the halides NaCl, NaBr, and NaI adds to the practical applicability, with low toxicity of estimated LD50 values of approximately 4000 (NaCl), 3500 (NaBr), and 4300 (NaI) mg/kg (rat, oral administration). This implies that halides might be suitable to adjust collagen tissues for specific uses in medicine, even if not all salt can be washed out completely prior to in vitro or in vivo use. The effects found in this preliminary investigation imply that the way of altering material properties tested here can become a valuable tool in tissue engineering in the future.

At the same time the application of the nanometer sized indenter at the tip of the AFM sensor may be regarded as a simulation of a nanoparticle that tries to enter into a collagen tissue.

To gain deeper insight in the mechanism and the quantity of the effects on different collagen tissues, quantitative measurements of denser and larger datasets with calibrated systems will clearly be of high value due to the high variability of artificial networks and natural tissues of collagen. Further research with additional salts, also ionic liquids, is suggested. Also reversibility and the extent to which a material modification by salt treatment remains after washing out the salt should be tested.

## Figures and Tables

**Table 1:** Viscoelastic properties of solvated collagen fibril and collagen molecules.

*Fibril*	Young's modulus (GPa)	0.43	*X-ray diffraction* (Sasaki and Odajima, 1996a)
	Average value 0.9	0.4–0.5	*MEMS stretching* (Eppell et al., 2006)
		0.2–0.5	*AFM testing* (Van Der Rijt et al., 2006)
		0.86 ± 0.45	*MEMS testing* (Shen et al., 2008)
		0.47 ± 0.41	*MEMS testing* (Shen et al., 2010)
		2.89 ± 0.23	*AFM testing* (Svensson et al., 2010a)
		1.87–1.94	*AFM testing* (Svensson et al., 2010b)
		0.3–1.2	*Atomisticmodeling* (Gautieri et al., 2011)
		0.12 ± 0.05	*MEMS testing* (Shen et al., 2011)
	Viscosity (GPa-s)	0.09–1.63	*MEMS testing* (Shen et al., 2011)
	Relaxation time (s)	7–102	*MEMS testing* (Shen et al., 2011)

*Molecule*	Young's modulus (GPa)	≈9	*Brillouin light scattering* (Harley et al., 1977)
	Average value 5.4 GPa	≈5.1	*Brillouin light scattering* (Cusack and Miller, 1979)
		3–5.1	*Estimate from persistence length* (Hofmann et al., 1984)
		2.9 ± 0.1	*X-ray diffraction* (Sasaki and Odajima, 1996b)
		0.35–12	*Optical trap* (Sun et al., 2002)
		≈7	*Reactive atomistic modeling* (Buehler, 2006)
		4.59 ± 0.38	*Atomistic modeling* (Gautieri et al., 2008)
		≈4	*Atomistic modeling* (Gautieri et al., 2009a)
		4.62 ± 0.41	*Coarse-grain modeling* (Gautieri et al., 2010)
		6–16	*Atomistic creep test* [present work]
	Viscosity (GPa-s)	(3.84 ± 0.38) · 10^−9^	*Atomistic creep test* [present work]
	Relaxation time (s)	≈0.5 · 10^−9^	*Atomistic creep test* [present work]

**Table 2:** Halide solution in the experiment.

Molarity (g/mol)	NaCl	NaBr	NaI
0.033	n.a.	n.a.	X
0.067	n.a.	n.a.	X
0.154	X	X	X
0.171	X	n.a.	n.a.
0.534	n.a.	n.a.	X
1.000	n.a.	n.a.	X
1.369	X	n.a.	X
